# Biochemical Storage Lesions Occurring in Nonirradiated and Irradiated Red Blood Cells: A Brief Review

**DOI:** 10.1155/2015/968302

**Published:** 2015-01-29

**Authors:** F. Adams, G. Bellairs, A. R. Bird, O. O. Oguntibeju

**Affiliations:** ^1^Training Department, Western Province Blood Transfusion Service, Cape Town 7450, South Africa; ^2^Western Province Blood Transfusion Service, Cape Town 7450, South Africa; ^3^Oxidative Stress Research Centre, Department of Biomedical Sciences, Faculty of Health and Wellness Sciences, Cape Peninsula University of Technology, Bellville, Cape Town 7535, South Africa

## Abstract

Red blood cells undergo a series of biochemical fluctuations during 35–42-day storage period at 1°C to 6°C. The sodium/potassium pump is immobilised causing a decrease in intracellular potassium with an increase in cytoplasmic sodium levels, glucose levels decline, and acidosis occurs as a result of low pH levels. The frailty of stored erythrocytes triggers the formation of haemoglobin-containing microparticles and the release of cell-free haemoglobin which may add to transfusion difficulties. Lipid peroxidation, oxidative stress to band 3 structures, and other morphological and structural molecular changes also occur leading to spheroechinocytes and osmotic fragility. These changes that transpire in the red cells during the storage period are referred to as “storage lesions.” It is well documented that gamma irradiation exacerbates storage lesions and the reports of increased potassium levels leading to adverse reactions observed in neonates and infants have been of particular concern. There are, however, remarkably few systematic studies comparing the *in vitro* storage lesions of irradiated and nonirradiated red cell concentrates and it has been suggested that the impact of storage lesions on leucocyte reduced red blood cell concentrate (RBCC) is incomplete. The review examines storage lesions in red blood cells and their adverse effects in reference to blood transfusion.

## 1. Introduction

Red blood cell transfusions are essential in the treatment of anaemia triggered by various pathologies or due to haemorrhage caused by trauma or surgery [1]. Globally, approximately 107 million units of whole blood were collected in 2013 of which 65% of blood transfusions in low-income countries were administered to children under 5 years of age while patients over 65 years comprised approximately 76% of transfusions in high-income countries [2]. While millions of whole blood and red blood cellular products are transfused annually, red blood cell concentrates (RBCCs) are still the most commonly transfused component.

There is a constant debate in the scientific community between individuals seeking to increase the time frame between donation and transfusion and those who are apprehensive regarding the efficacy and safety of stored blood. Although most clinicians agree that degeneration in blood and cellular components occur as soon as it is withdrawn from the donor's arm, patients requiring transfusions depend on the safety and efficacy of blood and blood components [3].

Since the First World War (1914–1918), technology has been available to store red cells under refrigerated conditions for short periods of time using sodium citrate as an anticoagulant. The advent of the Second World War (1939–1945) and the development of an anticoagulant containing an acid-citrate-dextrose (ACD) solution which significantly decreased the volume of anticoagulant required led to refrigerated blood being stored for 21 days and blood banking becoming a reality [4]. This allowed for increased volumes of blood to be transfused, a longer storage period, and a reduction in patients' experiencing citrate toxicity. Subsequently, further advances in the storage of red cell donations were made possible with the introduction of phosphates and adenine which allowed for a longer storage period of whole blood units. These advances encouraged the scientific community to develop additive solutions which would not only extend the storage period but also preserve the quality of the red cell concentrate during storage.

The introduction of component therapy where red cells are separated from plasma by centrifugation and the development of preservative solutions containing saline, mannitol, glucose, and adenine (e.g., SAGM) which were added to the separated erythrocytes, increased the storage period of red cell concentrates to 42 days when stored at 1°C to 6°C [5]. The addition of saline and mannitol decreases the haemolysis rate, and glucose provides an energy pathway substrate while adenine maintains the ATP levels. The standard RBC additive solution used in Europe is SAGM. In South Africa, the western coastal region uses SAGM, whereas the inland areas use ADSOL (a solution consisting of adenine, dextrose, sodium chloride, and mannitol). The blood transfusion establishments in America use AS-1 and AS-5 as additive solutions while the third additive solution to be licensed is AS-3. Although the additive solution AS-3, is a saline-adenine-glucose solution, it also contains phosphate and citrate as it is a SAGM variant. It should be noted that neither of the additive solutions has a major advantage over the other as fragmentation or vesiculation of the red cells still occurs in both solutions, although it has been reported that the membrane protein profile of RBCC stored in AS-3 seems to be better than that stored in SAGM [6].

Most customary blood bank practices involve the collection of approximately 450–575 mL of whole blood into a collection bag containing citrate-phosphate-dextrose (CPD) solution as the anticoagulant. A whole blood unit is not a commonly transfused product as the clotting factors and thrombocytes depreciate within hours of donation and therefore the expiry date is 35 days compared to the 42-day expiry date of other red blood cellular products. Whole blood is mainly used for patients suffering with massive haemorrhage or when neonatal exchange transfusions are needed [7]. Adult massive haemorrhage is defined as transfusion of more than 10 RBCC units within 24 hours which is approximately the total blood volume or replacement of more than 50% total blood volume using blood products within a 3-hour period.

Regardless of aetiology (surgical, trauma, or obstetrical aetiology), a hospital emergency patient undergoing massive haemorrhage requires the administration of a large volume of blood products within a short time to maintain haemostasis and satisfactory circulation as they are often admitted to hospital with multifactorial early trauma–induced coagulopathy (ETIC) which is associated with mortality. The hyperfibrinolysis and systemic anticoagulation observed in ETIC may be due to the tissue injury from surgery or trauma which leads to the local or systemic release of tissue factorcausing the activation of the coagulation pathways and it is this activation that causes a disseminated intravascular coagulation-like syndrome [8]. The resulting anaemia due to massive haemorrhage causes a reduction in primary haemostasis leading to platelet adhesion impairment and aggregation. The current management protocol used for hemorrhaging trauma patients is equal parts packed red blood cell concentrate, plasma, and platelets (i.e., 1 : 1 : 1 transfusion ratio), but this mixture of components is not whole blood as it also contains ±180 mL of added preservative solutions such as mannitol, dextrose, sodium phosphate, adenine, sodium bicarbonate, sodium chloride, and citric acid. While fresh whole blood also contains a preservative solution, the amount is less and the advantage of using this product compared to component therapy is the decreased total volume being transfused as well as the conservation of platelet function, all in one single unit [9].

In the processing laboratory, the unit of whole blood is centrifuged using a closed sterile system which results in maximal plasma removal. The major components are separated so that the different products may benefit multiple recipients. The additive solution, SAGM, is contained in an attached satellite bag and is added to the packed red blood cells (a red blood cell concentrate unit). The removal of the buffy layer reduces the presence of leucocytes by 70–80% from the original collection pack. The volume of the RBCC is approximately 300 mL, including the anticoagulant. In South Africa, RBCCs are normally prepared by removing the buffy layer. Clinical indications for transfusion of RBCC include acute blood loss of more than 30% of blood volume, anaemia, obstetric haemorrhage, or patients undergoing surgery. According to clinical trials examining the transfusion of red blood cell products, an RBCC transfusion is recommended when the haemoglobin level of the adult patient is less than 7 g/dL with a maintenance haemoglobin of between 7 to 9 g/dL, but this restrictive transfusion trigger is not necessarily applicable to all cardiac patients [10].

A guide regarding the indications for blood transfusions has constantly been under review since the advent of component therapy where the “10/30” (haemoglobin level maintained at 10 g/dL or haematocrit levels ≥30%) is maybe one of the oldest indications. Shander et al. provide a summary of current transfusion guidelines according to the American Association of Blood Banks, Society of Critical Care Medicine, the College of American Pathologists, and American Society of Anaesthesiologists, including the Society of Cardiovascular Anaesthesiologists, Society of Thoracic Surgeons, and the Italian Society of Transfusion Medicine and Immunohaematology. The similarities, albeit some of them questionable, indicate that transfusion should rather be implemented to escape ischaemia and improve patient general outcomes by limiting the number of allogeneic donor exposure instead of focusing on the maintenance of patient haemoglobin concentration levels to improve transfusion practices [11]. However, it has been reported that although these guidelines promotes best transfusion practice, it does not eliminate transfusion complications such as infectious and immunologic problems or adverse transfusion reactions causing mortality, for example, haemolytic transfusion reactions, transfusion circulatory overload, and transfusion related lung injury. Also, early reoperation due to extreme blood loss is associated with acute kidney failure, thrombotic embolism, myocardial infarction, and increased mortality and therefore best transfusion practice on its own may not be the best clinical practice [12].

The plasma and buffy layer (rich in thrombocytes, leucocytes, and reticulocytes) are subsequently extracted from the RBCC and separated into different, yet attached satellite bags. The buffy layer may be pooled and used to produce random donor platelet concentrates while the separated plasma may be used to produce fresh frozen plasma and cryoprecipitate or sent for fractionation to be processed into coagulation factor concentrates and plasma colloids such as albumin, stabilised human serum, or immunoglobulins [7].

It has been observed that ill infants requiring surgery or due to haemorrhage may have to be transfused with large quantities of stored red blood cell and transfusions of RBC containing increased levels of potassium have been associated with myocardial hyperkalaemia and neonatal arrhythmia [13, 14]. To reduce cost and wastage and to provide optimum benefit to neonates and infants, blood transfusion establishments produce the paediatric red blood cell concentrates (PRBCCs) and the infant red blood cell concentrates (IRBCCs). PRBCC is produced when a unit of red blood cells in additive solution is filtered through a leucocyte removal filter and equally divided between the SAGM bag and a transfer pack with a volume of 130 mL per unit while the IRBCCs are equally divided between 4 transfer bags after filtration with a volume of approximately 55 mL per unit. The additional transfer bag and leucocyte filter are sterile docked into the pack configuration. The IRBCC units are processed for neonates placed on the limited donor exposure program (LDEP) where multiple transfusions are expected in premature infants with low birth weights of less than 1500 g [15, 16].

The red blood cellular product may remain in the pack and used as RBCC or it may be further refined via leucocyte reduction using filtration methodology (a prestorage filtered RBCC unit). Many first world countries have adopted a universal leucocyte reduction (ULR) policy as filtered RBC products are believed to limit febrile nonhaemolytic transfusion reactions, prevent cytomegalovirus (CMV) transmission via transfusion, reduce postoperative infections, and reduce plasma haemolysis concentrations. In South Africa, however, selective use of these concentrates is recommended, as implementing the ULR policy would add significantly to the costs and there is still some controversy regarding some of the claimed benefits. South Africa has other main health concerns such as the escalating human immunodeficiency virus (HIV) pandemic and while the authors acknowledge the benefits of implementing a ULR policy, the cost of individual donation nucleic acid testing for HIV is immense, compared to the 4-, 8-, 16-, or 96-sample minipool NAT testing used in the United States. Also, leucocyte depletion may not inhibit the transmission of variant Creutzfeldt-Jakob disease to patients nor has the reactivation of viral infections (HIV and CMV) in standard nonleucocyte components been demonstrated [17]. Many of the filters used to deplete leucocytes from RBCC remove 3-4 log⁡^10^⁡ of white blood cells, but studies using rodents indicate that only 40–70% of the infectivity is removed and, as adequate infectivity remains, it may therefore become transmissible to a recipient [18]. A randomised clinical trial involving trauma patients demonstrated that there was no difference in infectious morbidity or mortality when transfused with prestorage leucocyte reduced RBCC compared to nonleucocyte reduced RBCC [19].

While there is extensive literature available regarding leucocyte reduced RBCC, there is less data available regarding the effects of gamma irradiation on prestorage leucocyte reduced red blood cellular concentrates. The American Food and Drug Association (FDA) recommends that a minimum of 75% recovery of transfused red blood cells must be present in the blood system 24 hours after transfusion [15]. Gamma irradiation exacerbates the storage lesion and the increased potassium levels over and above those seen in nonirradiated red blood cellular products due to the seepage of lactate dehydrogenase and potassium ions caused by irradiation exposure have been of particular concern. Gamma irradiation is indicated when a patient is at risk of developing transfusion-associated graft versus host disease (TA-GVHD) from being exposed to red blood cell components containing viable lymphocytes via transfusion [18]. TA-GVHD is a rare complication of transfusion and although it may be fatal, using gamma irradiation protects the vulnerable patients. The damaging effects of gamma irradiation on blood components are largely limited to red blood cells and do not significantly affect granulocyte and platelet function.

## 2. Biochemical Storage Lesions

It is well documented that certain biochemical changes occur during the 35 to 42 days of storing red blood cells at temperatures between 1°C and 6°C. The biochemical structure of the red blood cell (RBC) changes due to anaerobic glycolysis (cellular metabolism) and these changes are relative to the storage period.

### 2.1. pH

Ongoing glycolysis occurs when blood is stored in a plastic bag. Adenosine deaminase causes the breakdown of adenosine resulting in the formation of inosine and ammonia but is not regarded as clinically significant. An increase in protons causes the pH level to decrease and subsequently changes glycolytic metabolism. The decrease in pH causes the 2,3-diphosphoglycerate levels to decline with a simultaneous surge in adenotriphosphate (ATP) production. Glycolysis is slowed down and, as acid accumulates, the levels of ATP decrease and the shape of the red cell is gradually altered from discoid to echinocytic formations. This alteration in erythrocyte formation fades when stored blood is rejuvenated and is reversed when blood is warmed. The process of rejuvenation is when red blood cells are stored in a nutrient solution having a neutral pH [20]. The accumulation of lactic acid and proteins appear in the red cells after 14 days of storage due to glycolytic metabolism. It has been reported that a decrease in pH level and increases in lactate and potassium concentrations may occur within a few hours of storage while other changes may take weeks to appear [21].

### 2.2. 2,3 DPG

2,3-Diphosphoglycerate (2,3 DPG) is the enzyme regulator of haemoglobin and aids in oxygen transportation to the tissues of the body. The decrease in pH levels leads to an increase in 2,3 DPG degradation. This causes an increase in the oxygen affinity of haemoglobin leading to the oxygen dissociation curve shifting to the left, resulting in a reduction in oxygen to the peripheral tissues. In cases of hypoxia, the oxygen dissociation curve shifts the delivery to the right, thereby increasing oxygen transport to the tissues. After the 42-day storage period, a red blood cell unit may lose more than 90% of its 2,3 DPG concentration [5, 19, 22]. While 2,3 DPG levels may become undetectable within 2 weeks of storage, levels normalize within 72 hours after transfusion without any irreversible outcome observed and it is not considered clinically significant [21, 23].

Although multicountry guidelines recommend that RBC less than 5 days old be issued to patients undergoing massive transfusions, patients having RBC exchange procedures done, or patients in shock who are unable to increase their cardiac output, [7, 16, 17, 24] many retrospective trials demonstrate various conflicting outcomes including transfusion of older RBCC to be safer than fresh blood, no difference between transfusing fresh or older RBCC, or abnormal clinical conditions resulting from transfusing older blood. While it has been established that there is a definite difference between fresh and older blood, the clinical implication remains uncertain and therefore initiating prospective double-blind randomised clinical trials may resolve the ongoing debate [25]. A recent double-blind randomised clinical trial (the Age of Red Cells in Premature Infants {ARIPI}) compared the transfusion of fresh blood (mean storage of 5, 1 days) versus using older blood (mean storage of 14, 6 days) in premature neonates with birth weights less than 1250 g, to demonstrate the reduction of neonatal morbidities associated with organ failure or organ dysfunction as well as major nosocomial infections. The investigators concluded that there was no difference in clinical outcomes when transfusing premature, very low birth weight neonates with fresh blood compared to using older blood [26]. This study has, however, raised a few concerns regarding the implication that using older blood for transfusion does not affect necrotising enterocolitis, a common morbidity in premature infants. Also, a liberal transfusion practice was followed as haemoglobin levels were not stated prior to transfusion even though each infant received approximately 5 RBCC aliquots of about 14 mL per aliquot and thus the results of the trial may be challenging to establishments using a more conservative transfusion practice. Furthermore, the older blood used for neonatal transfusion had a mean storage time of 14, 6 days, whereas the average storage period of RBCC in American centres is about 18 days [27]. The aim of another randomised clinical trial was to determine whether the age of stored blood, used for transfusion, influences clinical outcomes in patients undergoing cardiac surgery. The results of the Red Cell Storage Duration Study (RECESS) demonstrated similar results to those of the ARIPI clinical trial. No differences regarding adverse transfusion reactions, changes in multiple-organ dysfunction scores, or mortality at day 28 were observed when using either fresh leucocyte reduced RBCC (stored for 10 days or less) or transfusing older RBCC (stored at 21 days or more) to cardiac patients requiring surgery [28].

### 2.3. ATP

The progressive loss of adenosine triphosphate (ATP) is well documented regarding morphological changes and RBC deformability during the storage period. ATP is not only an intracellular energy source but when ATP is released from the erythrocyte, it stimulates the production of nitric oxide leading to vasodilation during hypoxic conditions. The decrease of ATP concentration during storage causes the cellular reactions requiring energy, for example, phospholipid membrane distribution, active transport, and antioxidant reactions, to also decrease. It has been indicated that there is a 60% decrease in ATP levels after more than 5 weeks of storage [19]. The continuous reduction in ATP concentrations and acidification results in irreversible shape alteration of the RBC as echinocytic surface protrusions appear. The phospholipid bilayer loses its asymmetry and the shedding of microvesicles occur [20].

### 2.4. Potassium and Sodium Ions

Blood stored at 1° to 6°C decreases the rate of cellular metabolism and energy demand which allows blood to be stored for 35 to 42 days. This makes the sodium–potassium pump inoperative and consequently allows potassium ions to exit the cell and sodium ions to enter via the semipermeable membrane. It was demonstrated in critically ill patients that the sodium levels will revert to their normal levels within 24 hours after transfusion, whereas the potassium levels take about 4 days to stabilize [21, 22]. The extracellular potassium levels of stored blood increase daily at approximately 1 mEq/L. with the higher concentrations observed during the early days of storage [20]. Increased potassium levels in red blood cells may lead to arrhythmia when neonates or infants are transfused with large volumes of stored blood [14, 22].

### 2.5. Plasma Haemolysis

Due to a longer storage period, the red cell membrane experiences both biochemical and morphological alterations. These changes are referred to as storage lesions and such a biochemical indicator is plasma haemolysis or percentage haemolysis. Haemolysis of red blood cells (RBC) may occur during collection due to bacterial contamination, transportation, storage, donor red cell membrane deficiencies, presence of leucocytes in unfiltered RBC, mechanical injury during filtration process, or because of increased levels of vitamin C or penicillin in the donor [29].

The interaction of plasma haemoglobin with nitric oxide has been shown to cause endothelial dysfunction and is a risk factor for vasoconstriction, leucocyte adhesion, and intravascular thrombosis [30]. The release of hydrogen peroxidase and proteases by the leucocytes present in unfiltered blood may cause lysis of red blood cells during the storage period. Signs of haemolysis in the plasma or suspending fluid may suggest that the red blood cells have been either ruptured or it may be due to the loss of membrane-bound haemoglobin in microvesicles found on the cell's surface of intact cells. The addition of membrane stabilizers, for example, mannitol and citrate, may decrease haemolysis. It has been reported that, although the mean percentage haemolysis of RBCC stored in ADSOL (AS-1) was lower than its counterparts stored in SAGM, the difference was not statistically significant [31].

The easiest approach to assess the presence of haemolysis in a RBC unit before the unit is issued from the blood bank or prior to transfusion is by observation, but this visual inspection is often deceptive as it leads to an overestimation of haemolysis levels [32]. It has also been reported that pink/red discoloration due to haemolysis observed in either plasma or suspending fluid may often be due to plasma haemolysis levels being as low as 25 g/dL (±0, 09% plasma haemolysis) and under normal conditions these units are discarded unnecessarily. It is therefore advisable to incorporate a measure of quality control to determine plasma haemolysis accuracy by using either photometric or spectrophotometric methods on random units or prior to discarding the RBC unit [33].

The clinical implication of RBC haemolysis for the transfused individual is very serious and may lead to redox injury of the tissues, endothelium, or the proximal tubules of the kidneys while procoagulant and proinflammatory surfaces appear due to the infusion of microvesicles which affects the microcirculation and consequently impacts systemic haemodynamics [34]. Previous studies have indicated that patients with cardiovascular or circulatory pathologies should carefully consider using rheologically compromised RBC due to the haemodynamic risk [1, 23, 25, 34].

As the presence of haemolysis is a cause for concern, the guidelines dictated by the Council of Europe stipulate that the mean haemolysis level should be less than 0, 8%. The FDA has amended their standard regarding the mean haemolysis concentration by adding the “95/95 rule.” This rule states that, in addition to attaining the standard plasma haemolysis concentration of less than 1%, blood transfusion establishments must now demonstrate that 95% of their red blood cellular products meet the standard, statistically achieving 95% of the time [21, 35]. It is well documented that the concentration of haemolysis escalates during the storage period, but due to rigid quality control standards before, during, and after processing, together with trained personnel, the percentage haemolysis levels of most RBCCs do not exceed the prescribed limits [33, 34].

### 2.6. Leucocyte Reduced Red Blood Cells

Leucocytes found in red blood cellular allogeneic products are seldom of therapeutic benefit to the patient but are known to escalate the rate of cellular damage and to cause adverse transfusion reactions in recipients. These adverse reactions include alloimmunization to human leucocyte antigens (HLA), nonhaemolytic febrile transfusion reaction (NHFTR), transfusion-associated lung injury (TRALI), and immunomodulatory effects which include possible postoperation infection, postoperative mortality, or cancer recurrence [36, 37].

Leucocytes may also be regarded as the vector of infectious pathogens for instance Epstein Barr virus, cytomegalovirus and human T-lymphotropic virus I/II. It has been established that B-lymphocytes are vectors for the prions causing variant Creutzfeldt-Jakob disease [37]. It has been reported that using leucocyte reduced RBC reduces the incidence of multiorgan failure in patients having vascular or oncological surgery and decreases hospital stay by 2, 4 days as well as mortality in patients having gastrointestinal oncological surgery. The average reduction of 2, 4 days per patient would significantly cut costs of a national hospital [38]. British haemovigilance evidence demonstrates that using filtered RBC components reduce the frequency of transfusion-associated graft versus host disease.

It should be noted, however, that only using leucoreduced RBC to prevent TA-GvHD is not recommended as the RBC used for transfusion should be filtered and irradiated to prevent this serious and often fatal disease [39].

The filters used for leucocyte depletion are readily available and filtration of RBCC may be prepared at the patient's bedside during transfusion, before storage (in-line filtration) or after the buffy-coat layer and plasma have been removed (prestorage or 24-hour expiry product). Leucocyte depletion by filtration is best performed in the processing laboratory of the transfusion services as this maintains better quality assurance. It is advisable to filter the blood soon after collection and/or processing as granulocytes fragment and degranulate during storage, which may cause a NHFTR or the antigen-presenting cells presenting major histocompatibility complex (MHC) classes I and II antigens, leading to alloimmunization. It has been reported that leucocyte antibodies associated with TRALI are possibly targeted at HLA antigens (class II) and neutrophil alloantigens. In antibody-mediated TRALI, the antibody causing TRALI in a patient is usually recognised in multiparous female donors, but these donors cannot be excluded as this would reduce the donor-pool substantially [40]. The FDA recommends that a filtered unit of blood contains less than 5 × 10^6^ of white blood cells (WBC) and a retention of approximately 85% of the original RBC. Patients are stimulated to produce antibodies against the transfused histocompatibility antigens when the WBC exceed the 5-log count and thus to prevent primary alloimmunization, the FDA has stipulated this rule. They also suggest that quality control testing be done on 1% of filtered units, of which 100% should not have more than 5 × 10^6^ WBC.

Bedside filtration should be the last option to use as adequate quality control procedures cannot be completed on bedside leucocyte reduction filters. Bedside filtration requires a slow flow rate which reduces the filter performance. The filters that are currently used in most blood transfusion establishments provide a 3-log leucocyte depletion [41].

An increase in lactate dehydrogenase (LDH) concentration, glucose depletion, and haemolysis with a decrease in pH has been documented when buffy-coat-poor RBCC stored in SAGM was compared with its leucocyte depleted RBCC counterpart. This result shows that the presence of leucocytes in an RBCC unit was the source of the higher rate of haemolysis [37]. The reduction of leucocytes via filtration in red blood cellular products has not only minimized transfusion complications in patients exposed to allogeneic blood but has also decreased the occurrence of bacterial contamination, for example,* Yersinia enterocolitica* in red blood cellular components [23, 42], and a decrease in postoperational infections has been observed [35, 41].

As a result of the benefits, but despite the additional expense, leucocyte reduced RBCC has become the standard component for transfusion in many countries except in developing countries where it is not cost-effective. Germany had introduced filtered RBC as a primary component since 2001, whereas, in South Africa, selective use of these concentrates is recommended [17].

### 2.7. Irradiated Red Blood Cells

Despite reduced leucocyte concentration in the RBC units, a small amount of leucocytes remain in the pack and this usually does not pose a problem for patients with a healthy immune system unless they are receiving designated/directed donations. Red blood cellular products are irradiated to decrease the risk of transfusion-associated graft versus host disease (TA-GvHD) [43, 44].

TA-GvHD is a rare but fatal adverse transfusion reaction resulting from clonal proliferation and engraftment of viable donor T-lymphocytes and may occur in immunocompromised patients or in patients transfused with blood from donors who are homozygous for shared human leucocyte antigen (HLA) haplotypes. Gamma irradiation targets lymphocytic nucleic acids but also damages nonlymphoid cells in the process. There is a noticeable change in the properties of the RBC when they are irradiated. These alterations include lipid peroxidation due to reactive oxygen species (ROS), integrity of cell membrane is affected, seepage of potassium ions is accelerated, intracellular nucleotides are altered, and cell elasticity and deformability are decreased [39]. The literature reports increased plasma potassium, lactate dehydrogenase, and haemoglobin concentrations in irradiated red blood cellular products [43, 44]. Due to the storage lesions occurring during irradiation,* in vitro* haemolysis increases and, consequently, when irradiated RBC is transfused, the* in vivo *recovery is decreased, but this is not regarded as being clinically significant unless patients present with renal failure or the onset of hyperkalaemia. It has been reported that children are more susceptible to cardiovascular pathology due to hyperkalaemia than adults [45]. Neonatal hyperkalaemia may be prevented by using a cell saver to wash the irradiated RBC product for neonates undergoing cardiopulmonary bypass surgery [43]. It is also recommended that neonates at risk of hyperkalaemia requiring intrauterine or exchange transfusions be transfused within 24 hours after irradiation of RBC. Gamma irradiation of products is recommended for intrauterine transfusion, exchange transfusions, transfusions of first-degree relatives, premature neonates weighing less than 1200 g, patients with congenital immunodeficiency pathologies such as DiGeorge syndrome, Wiskott-Aldrich syndrome, all recipients of allogeneic bone marrow transplants, those undergoing stem cell harvesting for later autologous reinfusion, and patients who have received aggressive chemotherapy. Currently, gamma irradiation is the endorsed method for the prevention of TA-GvHD [46].

The American FDA and the Canadian guidelines have stipulated that the maximum storage period for irradiated RBC is 28 days; the Chinese State Food and Drug Administration (SFDA) has a ruling of 35 days [47], while the Council of Europerecommends that irradiation of nonleucocyte reduced RBC should not be completed for more than 14 days after donation and should not be stored for longer than 14 days after irradiation. Regarding irradiation dosage, the Council of Europe stipulates 25 Gy to 40 Gy on any given place on the bag, whereas the American FDA recommends not less than 15 Gy at any given place of the bag while 25 Gy must be delivered in the middle of the bag [39]. South Africa abides by the guidelines stipulated by the Council of Europe regarding expiry of irradiated RBC, but the irradiation dosage is between 25 and 50 Gy where the centre of the container is targeted [47].

The available data on the effects of irradiated red blood cellular products is limited as few systematic studies have been done, quality control testing is not performed on these units, and there is a variety of available guidelines regarding irradiation. Therefore, irradiated RBC storage lesions cannot be predicted.

## 3. Discussion

While blood transfusion establishments have certain guidelines specifying acceptable parameters regarding metabolic changes occurring in red blood cellular products during storage, the literature regarding its gamma irradiated counterparts is less extensive.

Many differences in the studies that evaluated the biochemical storage lesions and clinical impact of storage lesions in critically ill adult or paediatric patients were detected, for example, heterogeneous distribution (using fresh and old blood), preparation of blood products, study design, sample population, discrepancy in the differentiation of fresh and old blood, variety in irradiation guidelines, and different storage media used or preserving solutions not documented, small sample size and mostly limited to retrospective observations. It should also be noted that while it is common practice to use leucoreduced red blood cellular products in many first world countries, it is not a global practice.

## 4. Conclusion

The clinical impact that storage lesions have on RBC survival in transfused patients is debatable and therefore the evidence to substantiate the need for a shorter storage period is unsatisfactory. There are relatively few systematic studies comparing the* in vitro* metabolic changes occurring in irradiated red blood cellular concentrates and there is a need for randomised controlled trials to study the effect that storage lesions have on mortality and morbidity. Once these are underway, then perhaps this much debated question will be answered.

## 5. Recommendations

While the results of the RECESS and ARIPI clinical trials indicate that there are no differences regarding morbidity or mortality of transfusing patients using fresh blood compared to older blood, the occurrence of red blood cellular storage lesions may be influenced by the preparation of cellular components separated from the whole blood donation. Counties and transfusion establishments in different areas do not use the same anticoagulant solution such as SAGM, AS-3, CPD, or CPDA-1 and this may also influence the occurrence of storage lesions. Thus, the results attained from these trials should be carefully considered prior to implementing it as transfusion policy. Another randomised controlled trial of standard transfusion versus fresher red blood cell use in intensive care (TRANSFUSE) is currently ongoing and will determine whether using the freshest blood available for transfusion instead of using the standard older blood will reduce mortality in critically ill patients in intensive care units.

Considering the above reservations and inconsistencies observed regarding storage lesions occurring in nonirradiated and irradiated red blood cell components, it is recommended that more systematic research studies be initiated. Although prospective, randomised controlled trials may prove challenging due to storage lesion intricacy, diversity of patient pathophysiologies, and donor to donor variation, these studies should follow a similar pattern when investigating the* in vivo* effect of storage lesions in patient mortality and morbidity.
